# Portuguese validation of the Regret Intensity Scale (RIS-10) for measuring the intensity of regret associated with the provision of attention in health

**DOI:** 10.17533/udea.iee.v39n3e09

**Published:** 2021-11-08

**Authors:** Fabiana Rosa Neves Smiderle, Valmin Ramos-Silva, Stela Maris de Jezus Castro, Delphine Sophie Courvoisier, Rita Mattiello

**Affiliations:** 1 Enfermeira. Doutora. Professora da Escola Superior de Ciências da Santa Casa de Misericórdia de Vitória, EMESCAM, Espírito Santo, Brazil. Email: fabiana.neves@emescam.br Escola Superior de Ciências da Santa Casa de Misericórdia de Vitória Espírito Santo Brazil fabiana.neves@emescam.br; 2 Médico. Doutor. Professora. Escola Superior de Ciências da Santa Casa de Misericórdia de Vitória, EMESCAM, Espírito Santo, Brazil. Email: valmin.silva@gmail.com Escola Superior de Ciências da Santa Casa de Misericórdia de Vitória Escola Superior de Ciências da Santa Casa de Misericórdia de Vitória Espírito Santo Brazil valmin.silva@gmail.com; 3 Estatística. Doutora. Professora Universidade Federal do Rio Grande do Sul, Brazil. Email: stela.castro@ufrgs.br Universidade Federal do Rio Grande do Sul Universidade Federal do Rio Grande do Sul Brazil stela.castro@ufrgs.br; 4 Bioestatística. Doutora. Professora Hôpitaux Universitaires de Genève, Swiss. Email: delphine.courvoisier@hcuge.ch Hôpitaux Universitaires de Genève Swiss delphine.courvoisier@hcuge.ch; 5 Fisioterapeuta. Doutora. Professora Pontifícia Universidade Católica, Porto Alegre, Rio Grande do Sul, Brazil. Email: rita.mattiello@pucrs.br. Corresponding author Pontifícia Universidade Católica Porto Alegre Rio Grande do Sul Brazil rita.mattiello@pucrs.br

**Keywords:** emotions, health personnel, psychological adaptation, psychometrics, validation studies., emociones, personal de salud, adaptación psicológica, psicometría, estudio de validación., emoções, profissionais da saúde, enfrentamento, psicometria, estudos de validação

## Abstract

**Objective:**

The aim of the study was to adapt and validate the Regret Intensity Scale-10 (RIS-10) for Brazilian health professionals.

**Methods:**

The validation study took place in two phases, in which the first was the translation of the instruments and the second, the field validation using psychometric properties validity and reliability of the scale with 341 professionals (doctors, nurses and physiotherapists) linked to hospitals. Validity was assessed using content validities (six judges evaluation), criteria (correlation with the Life Satisfaction Scale - SWLS and Self-Reporting Questionnaire 20 -SRQ-20) and construct (exploratory analysis using the rotation method Promax, based on the slope graph and the Kaiser criterion and confirmatory using the structural equation model) after applying the questionnaire to professionals.Reliability was measured by Cronbach's α coefficient and retest test over a maximum period of 30 days. Reproducibility was calculated by intraclass correlation.

**Results:**

A total of 341 professionals participated, with an average age of 38.6 ± 9.2 years. The content validity index (CVI) was 1.00, for all items of the scale in the proportion of agreement of the judges. Exploratory factor analysis showed a satisfactory correlation (Kaiser-Meyer-Olkin = 0.88), suggesting a two-factor model, which comprises the main components of the emotion of regret (Factor I - emoticons, Factor II - feelings), accounting for 64% of the total variation of the first factor. In the confirmation, the index standardized root mean squared residual = 0.063 was close to the acceptable and other values were below. The scale correlated positively with SRQ-20 (*p* < 0.001) and negatively with SLWS (*p* = 0.003). Reliability showed (Cronbach’s α = 0.863) and test-retest reliability showed lower values ​​than expected. The Bland-Altman graph showed a mean bias of -1.5 with lower and upper limits of 15.8 to 12.8 respectively.

**Conclusion:**

The RIS-10 adapted for the population performed adequately in the psychometric properties evaluated for the assessment of the intensity of regret related to the provision of health care.

## Resumen

**Objetivo:**

Adaptar y validar la Escala de Intensidad de Arrepentimiento-10 (RIS-10) para profesionales de la salud brasileños.

**Métodos:**

Este estudio de validación se realizó en dos fases: la primera fue la traducción de los instrumentos y la segunda, la validación de campo evaluando las propiedades psicométricas de validez y confiabilidad de la escala con 341 profesionales (médicos, enfermeras y fisioterapeutas) vinculados a hospitales. La validez se evaluó mediante la validez de contenido (evaluación de seis jueces), criterios (correlación con la Escala de Satisfacción de Vida - SWLS y *Self-Reporting Questionnaire* 20 -SRQ-20) y constructo (análisis exploratorio mediante el método de rotación Promax, basado en el gráfico de pendiente (Criterio de Kaiser y confirmatorio por el modelo de ecuación estructural) luego de aplicar el cuestionario a los profesionales. La confiabilidad se midió mediante el coeficiente α de Cronbach y la prueba de reprueba en un período máximo de 30 días. La reproducibilidad se calculó por correlación intraclase.

**Resultados:**

Participaron 341 profesionales, con una edad media de 38.6 ± 9.2 años. El índice de validez de contenido (IVC) fue de 1.00 para todos los ítems de la escala en proporción de acuerdo con los jueces. El análisis factorial exploratorio mostró una correlación satisfactoria (Kaiser-Meyer-Olkin = 0.88), sugiriendo un modelo de dos factores, que comprende los componentes principales de la emoción de arrepentimiento (Factor I - emociones, Factor II - sentimientos), correspondiente al 64% de la variación total del primer factor. Tras la confirmación, el índice cuadrático medio residual estandarizado = 0.063 estuvo cerca de ser aceptable y los otros valores estaban por debajo. La escala se correlacionó positivamente con SRQ-20 (*p*<0.001) y negativamente con SLWS (*p* = 0.003). La confiabilidad mostró un α de Cronbach = 0.863 y la confiabilidad test-retest mostró valores más bajos de lo esperado. El gráfico de Bland-Altman mostró un sesgo medio de -1.5 con límites inferior y superior de 15.8 a 12.8, respectivamente.

**Conclusión:**

El RIS-10 adaptado a la población mostró un desempeño adecuado en las propiedades psicométricas utilizadas para evaluar la intensidad del arrepentimiento relacionado con la prestación de atención a la salud.

## Resumo

**Objetivo:**

O objetivo do estudo foi adaptar e validar a Regret Intensity Scale-10 (RIS-10) para profissionais de saúde brasileiros.

**Métodos:**

O estudo de validação ocorreu em duas fases, sendo a primeira a tradução dos instrumentos e a segunda, a validação de campo utilizando as propriedades psicométricas validade e confiabilidade da escala com 341 profissionais (médicos, enfermeiros e fisioterapeutas) vinculados a hospitais. A validade foi avaliada por meio de validades de conteúdo (avaliação de seis juízes), critérios (correlação com a Escala de Satisfação de Vida - SWLS e Self-Reporting Questionnaire 20 -SRQ-20) e construto (análise exploratória usando o método de rotação Promax, com base no gráfico de inclinação e critério de Kaiser e confirmatório pelo modelo de equações estruturais) após aplicação do questionário aos profissionais. A confiabilidade foi medida pelo coeficiente α de Cronbach e teste de reteste em um período máximo de 30 dias. A reprodutibilidade foi calculada por correlação intraclasse**.**

**Resultados:**

Participaram 341 profissionais, com média de idade de 38.6 ± 9.2 anos. O índice de validade de conteúdo (IVC) foi de 1,00, para todos os itens da escala na proporção de concordância dos juízes. A análise fatorial exploratória mostrou correlação satisfatória (Kaiser-Meyer-Olkin = 0.88), sugerindo um modelo de dois fatores, que compreende os principais componentes da emoção de arrependimento (Fator I - emoções, Fator II - sentimentos), correspondendo a 64% da variação total do primeiro fator. Na confirmação, o índice raiz quadrada média residual padronizada = 0.063 ficou próximo do aceitável e os demais valores ficaram abaixo. A escala correlacionou-se positivamente com SRQ-20 (*p* <0.001) e negativamente com SLWS (*p* = 0.003). A confiabilidade apresentou (α de Cronbach = 0.863) e a confiabilidade teste-reteste apresentou valores menores do que o esperado. O gráfico de Bland-Altman mostrou um viés médio de -1.5 com limites inferior e superior de 15.8 a 12.8, respectivamente**.**

**Conclusão:**

O RIS-10 adaptado para a população apresentou desempenho adequado nas propriedades psicométricas avaliadas para avaliação da intensidade do arrependimento relacionado à prestação de cuidados de saúde.

## Introduction

Health practice requires that, in addition to theoretical and practical knowledge, an emotional balance between practice and choices during activities emotional control psychological balance about their experiences. Acceptance by the professional that he cannot control all aspects of a situation is important for his mental health and, therefore, indirectly contributes to his quality of care.(1) Regrets related to clinical practice may be present at various moments of the professional-patient relationship, such as during diagnosis, treatment, evaluation of results, patient management, and interpersonal relationships.(2) The consequences of decisions made in a professional capacity can affect not only the clinical practice of professionals, but also their psychological and physical health.(1) Thus, a better understanding of feelings of regret experienced by health care practitioners and their consequences can contribute to improved emotional support and quality of care.(3)

Several instruments are capable of assessing the latent trait of regret in health professionals. However, these instruments do not evaluate regret comprehensively; more commonly, their scope is limited to the negative aspects of regret in a given situation.(4) Furthermore, some of the validated scales available in Brazil present an excessive number of items, which limits the use in most clinical scenarios.(5) In this context, the Regret Intensity Scale-10 (RIS-10), which comprises a mere 10 items, is a feasible scale that measures the self-reported intensity of feelings of regret related to care by health professionals. This instrument was originally developed in French and it was validated in German. The tool presented with good psychometric properties in both validations and presents a feasible approach for the screening of regret related to health practice. (1,6,7) Therefore, this study aimed to validate the RIS-10 in Brazilian health professionals.

## Methods

Study design and Participants. This cross-sectional study recruited from pediatric and adult populations in public and private hospital services in the states of Espírito Santo, Ceará, Pernambuco, Alagoas, Piauí, Bahia, Acre, Minas Gerais, Rio de Janeiro, São Paulo and Rio Grande do Sul from October 2018 to April 2019. Health professionals participated in the study (physicians, nurses, and physiotherapists), working in direct care to patients and who have at least six months of experience in the service. Participants were recruited through an invitation.

Data Measurements. (i) Sociodemographic variables were obtained through structured interviews and included age (years), sex (male or female), professional designations (title, number of works, work experience time, typical work shift, and state of origin); (ii) Regret Intensity Scale-10 (RIS-10) includes 10 items that assess the intensity of regret experience in the context of patient care within the last five years. The answer options ranged from 1 = no regret, to 5 = intense regret.(7) The intensity of regret is estimated by the total score, which is the sum of the responses of item on the scale, yielding a minimum score of 10 and a maximum of 50. The higher the score, the higher is the implied intensity of regret; (iii) Self-Reporting Questionnaire-20 (SRQ-20) was validated in Brazilian Portuguese. This tool comprises 20 items that propose to evaluate the prevalence of common mental disorders by evaluating depressive and anxious symptoms and somatic complaints.(8) The final score is the sum of the answers, which can range from 0 (null probability) to 20 (high probability); and (iv) Life Satisfaction Scale comprises five items answered using a 7-point Likert scale, with 1 = totally disagree, 2 = disagree, 3 = disagree slightly, 4 = neither agree nor disagree, 5 = agree slightly, 6 = agree, and 7 = totally agree.(9)

Validation. The RIS-10 questionnaire was validated in Brazilian Portuguese in two phases following the criteria proposed by the International Test Commission: Phase 1 - Instrument adaptation process and Phase 2 - Evaluation of the instrument's psychometric properties.(10,11)

### Phase 1 - Instrument adaptation process

Translation. Translation of the RIS-10 encompassed the following steps: (i) translation by two German-Brazilian Portuguese translators; (ii) harmonization between both Portuguese versions, resulting in a single version in Portuguese; (iii) back-translation of the harmonized version by two Brazilian Portuguese-German translators; (iv) harmonization between both translators, resulting in a single German version; and (v) general harmonization, where the versions resulting from the first and second harmonization were discussed by the four translators to obtain a consensus version.(10) We also translated the RIS-10 from French into Portuguese by two translators and harmonized these translations to assess the differences between the translated versions of German and French. Given that no differences were found between these translations, we adopted the German-to-Portuguese translation as the official translation.

### Phase 2 - Evaluation of the instrument's psychometric properties

Content validation. After the scale was translated, the process of cultural adaptation began. For this, this version of the scale was evaluated in relation to content by judges with clinical experience in the studied latent trait. Six judges who have been working in the health care area for more than 5 years participated from each of the following areas: 2 physicians, 2 nurses, 1 psychologist and 1 physiotherapist. First, the evaluation was done qualitatively, to obtain the possible suggestions for a better cultural adaptation of the translated terms. The level of agreement among the judges regarding the relevance and representativeness of the items was evaluated by the Content Validity Index (CVI). A 4-point Likert scale was used, where: 1 = not relevant; 2 = item needs a large revision to be representative (not relevant); 3 = quite clear, but needs a small review (very relevant); and 4 = quite clear and representative (highly relevant).(12) This index is calculated by the sum of the 3- and 4-point answers divided by the total number of judges, yielding a proportion of judges who deemed the item valid. However, 1- and 2-point answers required revision or elimination. To calculate the general CVI of the instrument, the sum of all CVI calculated separately was performed, divided by the number of items.(12) A CVI exceeding 0.78 is considered an acceptable agreement rate when six judges participate, which was the case in our study.(12) The scale’s content was evaluated through a pilot study of 10 professionals, six nurses, three physicians, and one physiotherapist.

Construct validity. Construct validity testing was performed with exploratory and confirmatory factor analysis. Exploratory factor analysis was performed with the Promax rotation method and used the Kaiser measure to assess the adequacy of the sample to a latent factorial structure. The evaluation of the adequacy of a latent factorial structure to the data was measured using the Kaiser-Meyer-Olkin (KMO) with polychoric correlation and the interpretation of the slope graph considered the number of factors corresponding to the change in the slope of the graph. Confirmatory factor analysis (CFA) verified the factorial structure suggested in the original scale with one factor using the structural equation model(7). The adjustment and quality of the sample of this study to the factorial structure was examined using the following: χ^2^ (chi-square model), goodness of fit index (GFI), root mean square error of approximation (RMSEA), standardized root mean squared residual (SRMR), normed-fit index (NFI), comparative-fit index (CFI), Tucker-Lewis index (TLI), and Bollen’s incremental fit index (IFI). The cut-off points considered acceptable for scale adjustment were as follows: χ^2^: *p* > 0.05,GFI > 0.90; RMSEA < 0.08, SRMSR < 0.10, NFI ≥ 0.90, CFI > 0.90, TLI > 0.95, and IFI > 0.90.(13)

Criterion validity. For criterion validity, the total score of the RIS-10 scale was correlated with the questionnaires validated in Brazil, namely, the SRQ-20 and the Life Satisfaction Scale. The intensity of regret is theoretically related to a higher prevalence of common mental disorders and lower life satisfaction. Correlations were evaluated using the Spearman’s rho (ρ), and values of *r* ˃ 0.3 were considered acceptable.(14)

Reliability. The reliability measures of internal consistency, floor and ceiling effects, test-retest, and Spearman-Brown coefficient were used. Cronbach’s α was used for internal consistency.(15) The floor and ceiling effect were evaluated by determining the lowest and highest percentage of the population in the application of the scale.(16) The Spearman-Brown coefficient was analyzed by the split method, as detailed in the following strategies. First, the items were randomly divided into two equal halves. A scale mean was computed for each half, and then the two sets of scale means were correlated to estimate a split-half correlation. The split-half correlation was adjusted by the Spearman-Brown formula to create a split-half reliability. (17) Test-retest reliability was analyzed using the intraclass correlation and Bland-Altman plots. Data collection for test-retest analysis was performed within a maximum period of 30 days. Interpretations of the reliability test items were as follows: Cronbach’s α was ≥0.7, as recommended;(15) the criterion considered to floor and ceiling effect was >20%;(16) the intraclass correlation (CIC) was considered acceptable when ≥0.7(15) and Spearman-Brown coefficient was >0.3.(14) The data were analyzed using the statistical software SAS v.9.4, the Lavann package v.0.6-5, and psych v.2.1.6 of R. This study uses a *p* of 0.05 as the statistical threshold of significance.

Sample size. Calculation of the sample size was based on the psychometric properties evaluated and aimed for a ratio of 10:1 (10 respondents for 1 item of the instrument). (18) Since the scale contains a total of 10 items, 100 participants would be needed.

Ethical issues. This study was approved by the ethics committee of the Pontifícia Universidade Católica do Rio Grande do Sul - PUC/RS (CAAE: 2.462.827/2018). All participants signed an informed consent form prior to the study. The use of the scale in this study was authorized by the author who developed it.

## Results

Sample characteristics. Considering the possible losses, we invited 500 professionals to participate in the study. Of the 500 total questionnaires distributed, 341 were completed (68%). Of the 159 questionnaires that were not returned, 119 were from the online version of the questionnaire 89 (75%) and 40 (25%) from the printed version. The proportion of participating institutions 9 public (64%), 3 private (21%) and 2 philanthropic (14%).

The mean age of the participants was 38.6 ± 9.2 years. The majority of the sample was female (217 of 341; 64%), and 190 (56%) respondents were married. Furthermore, 164 (48%) respondents were nursing professionals, one work only186 (56%) had only one employment relationship, and 135 (41%) worked the night shift. The interviewees originated predominantly from the state of Espírito Santo (76%; Table 1). The overall mean coping score was 2.3 ± 0.39.

**Table 1:** Characteristics of the Brazilian sample

Variables	***n*=341**
Age in years; mean (SD)	38.6 (9.2)
Sex; *n* (%)	
Male	124 (36)
Female	217 (64)
Marital status; *n* (%)	
Single	151 (44)
Married	190 (56)
Professional; *n* (%)	
Doctor	126 (37)
Nurse	164 (48)
Physical therapist	51 (15)
Amount of employment; *n* (%)	
One employment n (%)	186 (56)
Works at night shift; *n* (%)	135 (41)
State of origin; *n* (%)	
Espírito Santo	260 (76)
Rio Grande do Sul	38 (11)
Other	43 (13)

Instrument translation and cultural adaptation. The items of RIS-10 were consistent in both the translation and back-translation processes. Any terms that translated differently between translators were discussed and resolved to ensure uniformity of the instrument (Online supplement).

Content validity. The level of agreement among the judges regarding the relevance and representativeness of the items evaluated by the CVI was 1.00.

Construct validity. The exploratory factor analysis showed the adequacy and detection of the structure with KMO test (KMO = 0.88) and was considered a good sample fit for the latent factor structure. The analysis allowed the extraction of two factors, the first of which was responsible for 54% and with the second 64% of the total variation), as confirmed in the application of the slope graph. The correlation between the two factor was 0.75.

The factorial loadings of the latent factor structure are shown in Table 2. Items were distributed according to the structure suggested in the factor analysis composing a 2-factor model: Factor 1 comprises six items (3, 6, 7, 8, 9, and 10) of the scale, and Factor 2 was initially composed of four items (1, 2, 4, and 5). The factors describe the main components of the emotion of regret, which are feelings (i.e., emotions felt), physical manifestations, and cognitive processes. The lowest load item was “I feel undervalued”.

**Table 2:** Exploratory factor analysis with ProMax rotation factor loading for RIS-10

**Scale items**	Scale items in Portuguese	Factor I emotions	Factor II feelings
Q.8- I can’t concentrate right at work	Eu não consigo me concentrar direito no trabalho	0.876	-0.024
Q.7- I have trouble sleeping at home	Eu tenho dificuldades para dormir em casa	0.856	0.044
Q.10- I feel like crying	Eu tenho vontade de chorar	0.803	0.041
Q.9- I have the impression of no longer being made (the) for my profession	Eu tenho a impressão de não ser mais feita (o) para a minha profissão	0.643	0.042
Q.6- I get angry	Eu fico com raiva	0.574	0.180
Q.3- I feel devalued	Eu me sinto desvalorizado	0.422	0.271
Q.2- I feel uncomfortable	Eu me sinto mal	0.027	0.960
Q.1- Emotions come back to me	Eu tenho as mesmas emoções novamente	-0.064	0.752
Q.4- I feel ashamed	Eu sinto vergonha	0.182	0.635
Q.5- I have a knot in my stomach	Eu sinto um mal-estar no estômago	0.288	0.440
Eigenvalue		5.42	1.01

The CFA results were analyzed to verify the theoretical factorial structure: X2 = *p*<0.001), RMSEA = 0.114 (90% CI: 0.098-0.130), SRMR = 0.063, GFI = 0.894, NFI = 0.842, CFI = 0.866, TLI = 0.828, and IFI = 0.867. The SRMR performed close to acceptable in the sample of this study; however, according to the other adjustment measurements (GFI, NFI, CFI, TLI, and IFI), the factor solution was considered below acceptable.

Concurrent validity. The RIS-10 scale showed a moderated positive correlation with the SRQ-20 questionnaire (ρ = 0.40, *p* < 0.001) and negative correlation with the Satisfaction with Life Scale (ρ = -0.15, *p* < 0.003).

Reliability. The RIS-10 regret scale presented adequate internal consistency with Cronbach’s α coefficient (α = 0.86). Regarding the criterion of the floor and ceiling effects, values >20% were observed in the scale. The ground effect was found in nine of the 10 items that constitute the instrument (items 1, 3-10). The ceiling effect was only observed in item 2 (Table 3).

**Table 3:** Floor and ceiling effect of the RIS-10 scale

Scale Items	**Floor *n* (%)**	**Ceiling *n* (%)**	Average (SD)
1. Emotions come back to me	72 (21)	43 (13)	57.5 (20.5)
2. I feel uncomfortable	54 (16)	74 (22)	64 (14.1)
3. I feel devalued	142 (42)	37 (11)	89.5 (74.2)
4. I feel ashamed	129 (38)	52 (15)	90.5 (54.4)
5. I have a knot in my stomach	182 (53)	20 (6)	101 (114.5)
6. I get angry	152 (45)	31 (9)	91.5 (85.5)
7. I have trouble sleeping at home	200 (59)	23 (7)	111.5 (125.1)
8. I can't concentrate right at work	197(58)	20 (6)	108.5 (125.1)
9. I have the impression of no longer being made (the) for my profession	225 (66)	14 (4)	119.5 (149.1)
10. I feel like crying	192 (56)	21 (6)	106.5 (120.9)
Total = 10 items			94 (20.1)

**Figure 1:**
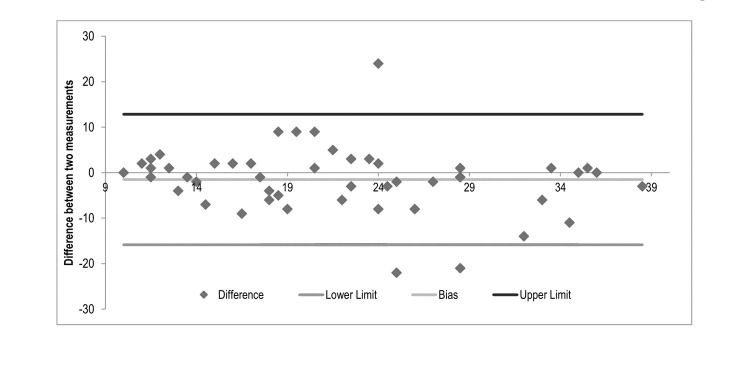
Bland-Altman graph of regret intensity (RIS-10) for baseline and 1-month follow-up surveys.

Eighty-seven professionals repeated the questionnaire for the test-retest reliability analysis. The intraclass correlation was 0.64 (95% CI: 0.5-0.75), and the Spearman-Brown coefficient ranged from 0.78 to 0.88 (SD = 0.05). Figure 1 shows the Bland-Altman plot of the agreement with the mean difference and the 95% agreement limits of the test and retest. The mean bias was -1.5, with lower and upper limits of 12.8 and 15.8, respectively.

## Discussion

The RIS-10 adapted for the Brazilian population presented with adequate psychometric properties, which may stem from how easily the questions were understood by the Brazilian population. The concise form of the questionnaire may also have contributed to its good psychometric performance. Likewise, the structured validation methodology and the input of professionals with different areas of expertise may have also played a contributory role.

The exploratory factor analysis suggested a two-factor structure, which differed from the original French and German versions that describe only a one-factor structure.(6,7) However, considering that nearly all of the total variance was explained by the first factor in the Brazilian version of the scale, one-factor structure was preserved. The items that diverged from the original version were: 1 = Emotions come back to me; 2 = I feel uncomfortable; 4 = I feel ashamed; and 5 = I have a knot in my stomach. Validity is not a fixed property and may differ according to population and situations.(19)

The intensity of regret assessed in the questionnaire was associated with consequences for mental health, due to the higher prevalence of common mental disorders such as depression and anxiety. Furthermore, and corroborating the results of the original study in French, intensity of regret, as measured by the scale, was found to be significantly related to lower satisfaction with life.(7) Exhaustion is strongly associated with affective-cognitive aspects, and there is evidence of its correlation with depression.(20) Decision regret may be associated with lower satisfaction, lower quality of life, lower levels of well-being, and other health problems such as anxiety, all of which can persist with the same intensity over time.(21,22)

Another important consideration is that our Brazilian scale showed a higher intensity of regret than did the German and French validation studies.(6,7) This discrepancy may have arisen from cultural differences, given that emotions are talked about more openly in Brazil than in the countries to which the scale has been validated. The German study, for instance, described the difficulty evinced by the interviewees at talking about their emotions6. Regret is valued more highly than is other emotions commonly deemed unpleasant and some people may be more affectively reactive than others, thereby influencing any measures of regret.(21) Some factors that contribute to decision-making conflict and to higher levels of regret include processing delays, low-quality decisions, or overestimated actions to reach the best possible decision.(21) Adopting a shared approach is considered essential not only to improve the quality of the decision, but also to minimize any undesirable consequences of regret on users and professionals.(23)

The reliability of the Brazilian adaptation, as determined by Cronbach’s alpha, was very close to that of the French (α =0.87) and German (α =0.88) versions,(6,15,24) considered sufficient according to the recommended parameters for internal consistency.(25) Unlike the German validation study, our study verified the ground effect with a 90% rate in relation to the responses at the lowest measurement levels. The reliability results of the RIS-10, accessed by the intraclass correlation, the Spearman-Brown coefficient, and Bland-Altman plot, were acceptable. These results can be explained by different intervals between the first and second test among professionals, completion of the questionnaire during their work shift, or other sources of error. There is no consensus in the literature on the ideal time interval between the first and second administration of the questionnaires;(19,26) however, it is recommended to be neither too short for the participant to have memorized the answers, nor too long that personal and environmental factors begin to interfere.(19)

Our study has limitations, one of which is the non-random sampling method that disproportionately represented the states of Espírito Santo and Porto Alegre. However, the study included participants from diverse states of Brazil (Southeast, Northeast, and South) that represent 83% of the population index and different areas of activity, thereby informing the validation of future instruments that can offer improved psychological services to health professionals throughout Brazil, given that most of the instruments are tailored for children and specific groups.(27) The study did not address professionals from institutions located in the states of the North and Midwest of the country.(28) However, we include the other regions and participants from public and private institutions for a larger representative population. Another contribution is attributed to the increase of scales validated for use in the health field with scope in the various scenarios of health professionals such as teaching, research, management, and clinical practice being a low-cost tool for its use.(29) The self-reporting methodology employed by questionnaires may be vulnerable to biases in self-esteem and social desirability. Nevertheless, questionnaires have the advantage of ease of administration over a wide range of potential scenarios. We did not evaluate the theory of response to the item, as used in the original study, due to the number of participants. A higher percentage of female respondents is observed, which can be attributed to the fact that demographic data in Brazil shows a predominance of women according to the annual population estimate from 2000 to 2060.(30) Also considering that in the health area there is a predominantly female contingent, mainly in the nursing team.(31) Other limitations include the restriction of the study population to health professionals in a hospital environment, and the limited generalizability to other professional environments in direct patient care. These limitations can help inform the design of future studies.

## Conclusion

The RIS-10 adapted for the Brazilian population presented with adequate psychometric properties as evaluated by health professionals. This scale appears to be a feasible, rapid, and easy to use tool for evaluations of regret in health professionals.

## Online supplement of Regret intensity scale (RIS-10) - Portuguese version

Até que ponto as afirmações a seguir aplicam-se a você **hoje** quando relembra esta situação da qual se arrependeu? (marcar um X na resposta adequada em cada linha)

*Quando penso na situação que mais me arrependo*... (1) De forma alguma a: (5) Com certeza

**Table:**

*Item*	*1*	*2*	*3*	*4*	*5*
Eu tenho as mesmas emoções novamente					
Eu me sinto mal					
Eu me sinto desvalorizado					
Eu sinto vergonha					
Eu sinto um mal-estar no estômago					
Eu fico com raiva					
Eu tenho dificuldades para dormir em casa					
Eu não consigo me concentrar direito no trabalho					
Eu tenho a impressão de não ser mais feita (o) para a minha profissão					
Eu tenho vontade de chorar					
